# An Introduction to the Special Issue “Mitochondrial Genome of Aquatic Animals: Analysis of Structure, Evolution and Diversity”

**DOI:** 10.3390/ijms26167871

**Published:** 2025-08-14

**Authors:** Yuri Phedorovich Kartavtsev

**Affiliations:** A.V. Zhirmunsky National Scientific Center of Marine Biology (NSCMB), Far Eastern Branch, Russian Academy of Sciences, 690041 Vladivostok, Russia; yuri.kartavtsev48@hotmail.com

The following Special Issue (SI) focuses on analyzing the structure, evolution, and diversity of mitochondrial genomes (mitogenomes) in predominantly aquatic animals, with two exceptions: semiaquatic lizards and the waterlily aphid. In line with the aims and scope of the *International Journal of Molecular Sciences (IJMS)*, submissions featuring biomolecular experiments were particularly encouraged. The scope of this SI was designed in alignment with current developments in molecular sciences and the expertise of its Guest Editors: the author of this Editorial and Dr. Ui Wook Hwang. In recent years, genomics (including mitogenomics) has emerged as a vast scientific frontier, with significant academic, social, and economic impacts driven by rapidly advancing technologies across molecular biology and related fields.

The editors searched for recent investigations within the scope of the SI’s primary topics, as defined above. Submissions were expected to address the following: the goals of the SI with respect to aquatic animals and their exceptional role in global ecosystems.

This collection comprises 13 original research articles, all presenting experimental studies that demonstrate the application of mitogenomes and other mitochondrial DNA (mtDNA) markers to address a range of issues in both basic biology and contemporary social problems. The contributions consider the structural and evolutionary dynamics of mitogenomes, phylogenetic informativeness, and diversity, functional aspects linked to organismal biology and lifestyle variation.

These topics fit the Special Issue’s framework. The initially proposed keywords remain fully applicable to the final publication set: aquatic animals, genomics, mitogenomes, structure, phylogenetic informativeness, evolution, comparative genomics, diversity, biomarker, and function.

In accordance with the publisher’s standard practice, manuscripts were submitted online through the MDPI platform (www.mdpi.com; accessed on 9 August 2025). Authors were required to register and log in to access the submission system, which could be reached via the designated submission link: [“Click here to submit your manuscript”]. As an international, peer-reviewed, open access journal published semimonthly by MDPI, *IJMS* provided all necessary services to the authors of the SI in line with the journal’s requirements, including access to MDPI’s professional English editing service, available both prior to initial submission and during the revision process.

In the first paper by Nikolaeva et al. (2023) [1], “Expression of Hairpin-Enriched Mitochondrial DNA in Two Hairworm Species (Nematomorpha)” (https://doi.org/10.3390/ijms241411411), the authors report on mtDNA in hairworms, a phylum of parasitic ecdysozoans best known for infecting arthropods.

In the paper by Li et al. (2023) [2], “The Complete Mitochondrial Genomes of Two Rock Scallops (Bivalvia: Spondylidae) Indicate Extensive Gene Rearrangements and Adaptive Evolution Compared with Pectinidae” (https://doi.org/10.3390/ijms241813844), wide gene rearrangements and apparent adaptive evolution are studied and compared with those of other scallops in the family Pectinidae.

In the paper by Zhou et al. (2024) [3], “The Complete Mitochondrial Genome and Phylogenetic Analysis of the Freshwater Shellfish *Novaculina chinensis* (Bivalvia: Pharidae)” (https://doi.org/10.3390/ijms25010067), poorly studied but ecologically and economically significant razor clams (Pharidae and Solenidae) are studied.

In investigations by Kundu et al. (2024) [4], “Mitogenomic Characterization and Phylogenetic Placement of African Hind, *Cephalopholis taeniops*: Shedding Light on the Evolution of Groupers (Serranidae: Epinephelinae)” (https://doi.org/10.3390/ijms25031822), the authors clarify the mitogenome structure and some of its functions in the evolution of groupers (Serranidae).

In the study by Tao et al. (2024) [5], “Comparative Mitogenome Analyses of Fifteen Ramshorn Snails and Insights into the Phylogeny of *Planorbidae* (Gastropoda: Hygrophila)” (https://doi.org/10.3390/ijms25042279), the authors focus on the phylogeny of Ramshorn snails, which play crucial ecological roles as low-trophic-level organisms and vectors for zoonotic trematodes.

In the paper by Albasaud et al. (2024) [6], “Complete Mitochondrial Genomes and Phylogenetic Analysis of Genus *Henricia* (Asteroidea: Spinulosida: Echinasteridae)” (https://doi.org/10.3390/ijms25115575), the authors provide mitogenomic insights that resolve some taxonomic uncertainties in the morphologically variable sea stars of the genus *Henricia*.

In the paper by Hong et al. (2024) [7], “Differential Mitochondrial Genome Expression of Four Hylid Frog Species under Low-Temperature Stress and Its Relationship with Amphibian Temperature Adaptation” (https://doi.org/10.3390/ijms25115967), the genomic expression of a hylid frog species is studied under cold stress conditions and its climate adaptation is elucidated.

In the paper by Kartavtsev and Masalkova (2024) [8] “Structure, Evolution, and Mitochondrial Genome Analysis of Mussel Species (Bivalvia, Mytilidae)” (https://doi.org/10.3390/ijms25136902), the authors report on one of the most abundant bivalves, the mussel family Mytilidaeprovides, and discuss its mitochondrial structure, evolution, and the effect of natural selection on its molecular polymorphisms in nature.

In the study by Wei et al. (2024) [9], “The Mitogenomic Landscape of Hexacorallia Corals: Insight into Their Slow Evolution” (https://doi.org/10.3390/ijms25158218), the authors highlight why mitogenomes have become powerful tools for reconstructing animal evolutionary history.

The paper by Zhan et al. (2024) [10], “The Phylogenetic Relationships of Major Lizard Families Using Mitochondrial Genomes and Selection Pressure Analyses in Anguimorpha” (https://doi.org/10.3390/ijms25158464), provides insights into limb loss adaptation and phylogenetic relationships in Squamate reptiles inferred from a comparative analysis of mitogenome sequences.

In the study by Shi et al. (2024) [11] “Characterization, Codon Usage Pattern and Phylogenetic Implications of the Waterlily Aphid *Rhopalosiphum nymphaeae* (Hemiptera: Aphididae) Mitochondrial Genome” (https://doi.org/10.3390/ijms252111336) the codon usage and phylogeny of waterlily aphid were analyzed.

In the paper by Ye et al. (2024) [12], “Potential Cryptic Diversity in the Genus Scoliodon (Carcharhiniformes: Carcharhinidae): Insights from Mitochondrial Genome Sequencing” (https://doi.org/10.3390/ijms252111851), the mitogenome of a shark species was determined and used to resolve some phylogenetic relationships.

Lastly, in the paper by Nikolaeva et al. (2025) [13], “Rare Evolutionary Events Support the Phylogenetic Placement of Orthonectida Within Annelida” (https://doi.org/10.3390/ijms26135983), the authors examine the phylogenetic placement of orthonectids using an approach that takes into account rare evolutionary events with an assessment of their probabilities based on a reference sequence database (RefSeq, NCBI). This approach provides compelling evidence of the branching of orthonectids among annelids but does not conclusively resolve their position among the annelid taxa. 

The Guest Editors of this SI would like to thank the authors for their contribution to a collection of papers that underscore the value of investigations into the structure, evolution, and diversity of mitogenomes across aquatic animals. The collected works have made significant contributions to three key areas:

1. Molecular Phylogenetics—Emerging as the most developed field, with multiple papers advancing fundamental concepts in evolutionary relationships;
2. Structural Genomics—Detailed examinations of mitogenome organization and variability;
3. Adaptive Evolution—Analyses of selection pressures shaping mitochondrial DNA.

The main goals of this SI were thoroughly achieved within its framework. All topics, such as the structure, evolution, and diversity of the mitogenome in aquatic animals, were considered. The only gap that remains unresolved is mitonuclear relations. There is thus room for *IJMS* to report on further developments in this field of study. In addition, there is a concern regarding our overconfidence in the detection of the impact of natural selection at the level of single markers. Investigations into the effects of natural selection on molecular markers, including those presented in this SI, are reported frequently; however, little attention is paid to the many complexities within the field, as stressed in the study by Kartavtsev et al. [8]. The most successful subjects developed in this SI are molecular phylogenetics and phylogenomics. Each paper addresses these topics comprehensively, and certain fundamental generalizations appear in several papers included in this SI. The editors believe that the readers will appreciate the work presented in this Special Issue.
